# *N*‐[2‐(4‐benzoyl‐1‐piperazinyl)phenyl]‐2‐(4‐chlorophenoxy) acetamide is a novel inhibitor of resorptive bone loss in mice

**DOI:** 10.1111/jcmm.16228

**Published:** 2020-12-23

**Authors:** Zhihao Chen, Eunjin Cho, Mina Ding, Jihyoun Seong, Xiangguo Che, Sunwoo Lee, Byung‐Ju Park, Je‐Yong Choi, Tae‐Hoon Lee

**Affiliations:** ^1^ Department of Molecular Medicine Chonnam National University Graduate School Gwangju Korea; ^2^ Department of Oral Biochemistry Dental Science Research Institute Korea Mouse Phenotyping Center School of Dentistry Chonnam National University Gwangju Korea; ^3^ Department of Biochemistry and Cell Biology Cell and Matrix Research Institute Korea Mouse Phenotyping Center KNU Convergence Educational Program of Biomedical Sciences for Creative Future Talents School of Medicine Kyungpook National University Daegu Korea; ^4^ Department of Chemistry Chonnam National University Gwangju Korea

**Keywords:** acetamide, bone resorption, osteoclastogenesis, piperazine

## Abstract

The dynamic balance between bone formation and bone resorption is vital for the retention of bone mass. The abnormal activation of osteoclasts, unique cells that degrade the bone matrix, may result in many bone diseases such as osteoporosis. Osteoporosis, a bone metabolism disease, occurs when extreme osteoclast‐mediated bone resorption outstrips osteoblast‐related bone synthesis. Therefore, it is of great interest to identify agents that can regulate the activity of osteoclasts and prevent bone loss‐induced bone diseases. In this study, we found that *N*‐[2‐(4‐benzoyl‐1‐piperazinyl)phenyl]‐2‐(4‐chlorophenoxy) acetamide (PPOAC‐Bz) exerted a strong inhibitory effect on osteoclastogenesis. PPOAC‐Bz altered the mRNA expressions of several osteoclast‐specific marker genes and blocked the formation of mature osteoclasts, suppressing F‐actin belt formation and bone resorption activity in vitro. In addition, PPOAC‐Bz prevented OVX‐induced bone loss in vivo. These findings highlighted the potential of PPOAC‐Bz as a prospective drug for the treatment of osteolytic disorders.

## 1 INTRODUCTION

Bone plays many vital roles in our body: it provides mechanical support for movement; protects the brain, bone marrow and other vital organs; and functions as an endocrine organ to modulate energy expenditure.^1^ These functions are involved in continuous bone remodelling.^2^ In bone remodelling, old bone is resorbed and replaced by newly synthesized bone, which is governed by bone‐resorbing cells (osteoclasts) and bone‐forming cells (osteoblasts).^3^, ^4^ During normal bone remodelling, bone formation should be closely integrated with bone absorption to maintain a dynamic balance to avoid excessive loss in bone mass.^2^ In many pathological conditions, bone formation is exceeded by osteoclast‐mediated bone resorption, which results in excessive bone loss.^5^ Osteoporosis, a common bone disease caused by the excessive formation of osteoclasts and increased resorption activity, results in a decrease in oestrogen levels.^6^ Changes in osteoclast formation and overactive resorption activity also contribute to the bone destruction that occurs in osteoporosis and to osteolysis‐mediated osteolytic complications of metastatic tumours such as breast cancer.^7^

A recent review summarized the findings related to osteoclast regulation by several cytokines, including receptor activator of nuclear facto‐kappa B (RANK), receptor activator of nuclear factor‐kappa B ligand (RANKL), macrophage colony‐stimulating factor (M‐CSF), osteoprotegerin (OPG), interleukin‐1 (IL‐1), tumour necrosis factor (TNF) and interleukin‐1 (IL‐6).^8^ Of these, the interaction between RANKL and RANK is one of the most popular review topics.^8^ RANKL, a member of the TNF superfamily, stimulates the differentiation of osteoclast precursor cells into osteoclasts.^9^ Marrow stromal cells and osteoblasts can produce RANKL, which has been suggested to correlate with the activation of osteoclast differentiation.^10^ RANK, the receptor for RANKL, is also a TNF receptor superfamily member located on the osteoclast precursor and mature osteoclast cell surface.^9^ In addition to RANKL, M‐CSF also has a critical function in osteoclast formation. M‐CSF can induce osteoclast differentiation from osteoclast precursors and prolong the survival of mature osteoclasts.^11^ In addition, M‐CSF serves as a potent stimulator for the induction of RANK expression in osteoclast precursor cells.^12^ After RANKL binds with RANK, the tumour necrosis factor receptor ‐associated factor 6 (TRAF6) is recruited, which is capable of activating RANKL‐mediated signalling pathways through autoactivation and trimerization and subsequently inducing the phosphatidylinositol 3‐kinase/protein kinase B (PI3K/Akt), nuclear factor‐kappa B (NFκB) and mitogen‐activated protein kinases (MAPKs) signalling pathways.^13^ Following their activation, nuclear factor of activated T‐cell cytoplasmic 1 (NFATc1), the main transcription factor of osteoclasts, can be induced, which is able to trigger the expression of osteoclast‐related genes such as cathepsin K (*CtsK*), and matrix metalloproteinase‐9 (*MMP‐9*).^14^, ^15^, ^16^ The currently available and most popular clinical drugs for the treatment of bone loss‐related diseases are bisphosphonate and its derivatives.^17^, ^18^ However, their treatment is always associated with side effects such as jaw and hypocalcaemia osteonecrosis,^17^, ^18^ which emphasizes the need for the discovery of new preventive drugs for the treatment of bone loss‐related diseases.

To identify candidate disease‐modifying osteoporosis drugs (DMOPDs), an initial screen was performed of our in‐house‐synthesized compounds (52 synthetic small molecule compounds) using a TRAP staining assay; subsequently, PPOAC‐Bz was identified as a strong inhibitor of osteoclastogenesis. After examining the molecular mechanisms underlying the suppressive effects of PPOAC‐Bz during osteoclastogenesis in vitro, we found that PPOAC‐Bz repressed the RANKL‐mediated formation of osteoclasts in the early stage. In addition, PPOAC‐Bz inhibited the formation of mature osteoclasts, subsequently attenuating bone resorption activity during osteoclastogenesis. In vivo, PPOAC‐Bz can lead to the prevention of ovariotomy (OVX)‐induced bone loss. Collectively, these results suggested that PPOAC‐Bz inhibited the formation of osteoclasts in vitro and blocked bone loss in vivo, highlighting its potential for the treatment of bone resorption‐related diseases.

## 2 MATERIALS AND METHODS

### 2.1 Reagents and antibodies

PPOAC‐Bz was obtained from ChemBridge (San Diego, CA, USA) and dissolved in dimethyl sulfoxide (DMSO; D2650, Sigma‐Aldrich, St. Louis, MO, USA) before use. Alpha‐modified minimal essential medium (α‐MEM; 12561‐056) and foetal bovine serum (FBS; GB0163) were obtained from Thermo Fisher Scientific (Waltham, MA, USA). RANKL (315‐11‐500 µg) and M‐CSF (315‐02‐500 µg) were procured from PeproTech (Rocky Hill, NJ 08553, USA). The TRAP staining assay kit (AKO4F) and bone resorption assay kit (CSR‐BRA‐48×2) were purchased from Cosmo Bio Co., Ltd. (Tokyo, Japan). Anti‐β‐actin (A5441; Sigma‐Aldrich, St Louis, MO, USA), anti‐cathepsin K (48353) and anti‐c‐Src (sc‐8056) were from Santa Cruz Biotechnology, Inc (Dallas, TX, USA); anti‐c‐fos (4384s), anti‐phospho‐p65 (s536), anti‐phospho‐p38 (4511s), anti‐phospho‐ERK1/2 (4370s), anti‐phospho‐JNK (9255s), anti‐phospho‐AKT (4060s), anti‐phospho‐IκBa (2859s), anti‐p65 (4764s), anti‐p38 (9212s), anti‐ERK1/2 (9102s), anti‐JNK (9252s), anti‐AKT (9272s), anti‐IκBa (9242s), anti‐NFATc1 (8032s) and horseradish peroxidase (HRP)‐conjugated secondary antibodies (7074S) were supplied by Cell Signaling Technology (Boston, MA, USA). The electrochemiluminescence (ECL) system (RPN2106) for the detection of chemiluminescence signals was from iNtRON (Seoul, Korea). The BCA protein assay kit (23225) was purchased from Pierce Biotechnology (Rockford, IL, USA).

### 2.2 Bone marrow‐derived macrophage isolation and culture

For the in vitro osteoclastogenesis assay, mice bone marrow‐derived macrophages (BMMs) were collected as described previously.^13^, ^19^ Briefly, 10‐week‐old C57BL/6J mice were killed in a CO_2_ filled box, and BMMs were isolated from the mice tibiae and femur bone via flushing the bone marrow using α‐MEM. The flushed cells were collected and cultured in α‐MEM supplied with 10% heat‐inactivated FBS and 1% penicillin/streptomycin. On the morning of the second day, the non‐adherent cells were collected, cultured in a Petri dish, and treated with M‐CSF (30 ng/mL) to select the BMMs. After incubation in a cell culture incubator for 3 days, the adherent cells (BMMs) were detached using a cell‐free enzyme and collected. The collected cells were further cultured in induction medium to induce osteoclast differentiation. The IACUC at Chonnam National University approved all the animal experiments (approval number: CNU IACUC‐YB‐2019‐46).

### 2.3 In vitro osteoclastogenesis and cell viability assay

BMMs (2 × 10^4^ cells per well) were cultured in 48‐well plates and treated with 30 ng/mL M‐CSF and 50 ng/mL RANKL until the formation of mature osteoclasts was observed in the DMSO treatment group. Next, the osteoclasts were fixed in 4.0% formaldehyde (BP031) for at least 15 minutes and stained using a TRAP staining kit. The spread of the osteoclast area and the formed osteoclast numbers were counted using ImageJ software (NIH, Bethesda, MD) on the basis of the number of nuclei (n ≥ 3) visible under a microscope.

The viability of the BMMs was assessed after incubation with PPOAC‐Bz using a cell viability assay kit. BMMs (1 × 10^4^ cells/well) were cultured overnight in a 96‐well plate. After RANKL, M‐CSF and various concentrations of PPOAC‐Bz were added to the cells, the cells were incubated at 37°C in 5% CO_2_. After 24 or 72 hours, the medium was replaced with FBS‐free medium containing 10% of cell viability reagent. The cells were incubated at 37°C in 5% CO_2_ for a further 30 minutes, and the absorbance of each well at 450 nm was measured using a SpectraMax i3x microplate reader (Molecular Devices, San Jose, CA, USA).

### 2.4 Screening of small compound libraries

Libraries containing 52 synthetic small molecule compounds were obtained from ChemBridge (San Diego, CA, USA). Our primary screening method was image‐based, using BMMs, and was performed in a 96‐well plate format. BMMs were treated with each molecule at 10 µmol/L or with DMSO for 3 days to allow the formation of mature osteoclasts with RANKL and M‐CSF treatment by completely replacing the medium every other day. After mature osteoclasts formed in the control group, all cells were assessed using the TRAP staining assay. After air‐drying for 2 days, the area of the matured osteoclasts in each group was counted using ImageJ. If the average of the total differentiated cell areas was less than that in the control group, the compounds were regarded as inhibitors of osteoclastogenesis.

### 2.5 Bone resorption and F‐actin belt immunofluorescence assay

A bone resorption assay kit was used to evaluate the osteoclast bone resorption activity in accordance with the manufacturer's instructions. BMMs (2 × 10^4^ cells/well) were cultured in the kit‐supplied coated‐plate with M‐CSF supplementation. On the following day, the medium was replaced, and the cells were incubated with M‐CSF and RANKL and treated with or without the indicated concentrations of PPOAC‐Bz until the formation of mature osteoclasts was observed. On the following day, the supernatant in each well was harvested into a black polypropylene 96‐well microplate (30496; Thermo Scientific Nunc) and mixed with NaOH (S5881). Subsequently, the fluorescence intensity of each well was measured using the SpectraMax i3x fluorescence plate reader (excitation wavelength: 485 nm; emission wavelength: 535 nm). The resorptive area was calculated based on 10 randomly selected pictures per well using the ImageJ software, as previously described.^13^, ^19^

The F‐actin belts of the osteoclasts were detected using a rhodamine‐conjugated phalloidin staining assay (A12379; Thermo Fisher Scientific).^20^ BMMs were seeded on 12‐mm cover slips in the presence or absence of PPOAC‐Bz and then treated with M‐CSF and RANKL. After formation, the osteoclasts were fixed for at least 15 minutes in 4.0% paraformaldehyde and then blocked via incubation in 5% FBS for 60 minutes. After washing using PBS, rhodamine‐conjugated phalloidin (1:40) was added into each well to visualize the F‐actin belts. After 20 minutes, a 1:1500 solution of DAPI was added to the cells for 5 minutes. The cells were then washed three times with PBS and observed under a fluorescence microscope.

### 2.6 RNA isolation and quantitative real‐time PCR

BMMs were cultured in a 6‐well plate in the presence or absence of PPOAC‐Bz for 4 days in the induction medium. The total RNA from the BMMs was isolated using a QIAzol RNA lysis reagent (15596018; Qiagen Sciences, Valencia, CA, USA) as described in our previous study.^13^ A PrimeScript™ RT reagent kit for qRT‐PCR (RR420A; Takara Biotechnology, Tokyo, Japan) was used to synthesize cDNA in accordance with the manufacturer's protocol, and real‐time PCR was performed with a QuantStudio 3 qRT‐PCR system (Applied Biosystems, Foster City, CA, USA) together with the Power SYBR Green PCR Master Mix (4367659; Applied Biosystems, Foster City, CA, USA) and a temperature protocol provided by the company.^13^, ^19^, ^21^ The cycle threshold values obtained were expressed as relative ratios and calculated using the 2^−ΔΔ^ CT method; the expression levels of the mRNA were normalized to the glyceraldehyde 3‐phosphate dehydrogenase (GAPDH) expression, as reported in our previous study.^13^, ^19^, ^21^ The primers used for real‐time PCR assay are listed in Table S1.

### 2.7 Western blot assays

Osteoclasts were lysed using a RIPA buffer (89900; Thermo Fisher Scientific). After lysis, the cells were centrifuged at 16 400 *g* for 30 minutes at 4°C; the pellets were discarded, and the supernatant was retained. The concentration of protein in the supernatant was measured using a BCA protein assay, and samples with equal protein concentrations were boiled and electrophoresed on a 12% SDS‐PAGE gel. The separated proteins were transferred to PVDF membranes. After non‐specific binding to the membrane was blocked via the incubation of the membrane in 5% skim milk for 1 hour, the membranes were incubated overnight with the appropriate primary antibodies (at a 1:1000 dilution) at 4°C. After three washes with TBST, the membrane was incubated at room temperature for 1 hour with HRP‐conjugated secondary antibodies (at a 1:2000 dilution), and an ECL reagent was applied to detect the chemiluminescence signals, in accordance with the manufacturer's protocol.

### 2.8 OVX‐induced osteoporosis mouse model

The mice were housed in a specific pathogen‐free facility. To evaluate the effect of PPOAC‐Bz on osteoclastogenesis in vivo, we developed an OVX‐induced bone loss model. Briefly, 30 healthy 7‐week‐old C57BL/6 female mice were divided into three treatment groups (sham, control and treatment). The mice in the control and treatment groups received an OVX; the sham group mice only received an abdominal incision. After recovery for 1 week, the treatment group mice were injected intraperitoneally with PPOAC‐Bz (20 mg/kg), prepared as a solution in 5% Tween 80 and 5% DMSO, every other day for 5 weeks. The sham and control groups received an equal volume of a mixture of 5% Tween 80 and 5% DMSO. The mice were weighed every week. On the final experimental day, the mice were killed, serum was collected for biochemical analysis, and both the femurs and tibias were fixed in 3.7% paraformaldehyde after removal of the surrounding tissues for further microcomputed tomography (micro‐CT) and histological functional analysis.

### 2.9 Microcomputed tomography (micro‐CT) scanning and histological functional analysis

A Quantum GX Micro‐CT imaging system (PerkinElmer, Hopkinton, MA, USA) at the Korea Basic Science Institute (Gwangju, Korea), was used for performing the CT imaging. The fixed tibias and femurs were analysed using a high‐resolution micro‐CT instrument; the mean of the bone surface density (BS/BV), mean of the trabecular volume (Tb.V), trabecular bone volume per total volume (BV/TV), mean of pore number (Po.N), mean of pore density (Po.Dn) and mean of the bone mineral density (BMD) were measured. Following micro‐CT analysis, the femurs were decalcified in 20% EDTA (Sigma‐Aldrich) at 4°C for 5 days and then embedded in paraffin to prepare sections for further functional analysis. Subsequently, the samples were deparaffinized, and haematoxylin and eosin (H&E) staining and TRAP staining were performed.

### 2.10 Three‐point bending test

The right femur of each mouse was removed and wrapped in 0.9% NaCl‐soaked gauze and then stored at −20°C. The femurs were rehydrated overnight in 0.9% NaCl at 4°C before analysis. Mouse femurs were set on the applicable mould, and the pressure sensor was set at the maximal allowable distance for each bone without compromising the test (20.0 mm for the femur). The three‐point bending test was performed with a miniature materials testing machine (Instron, MA, USA). The crosshead speed descent during testing was 1 mm/min, and the force‐displacement data were collected as the maximum load and slope of the bones.

### 2.11 Serum biochemical analysis

The serum calcium and phosphorus levels were analysed using commercially available kits (Bio Assay Systems). For serum calcium analysis, 5 μL of serum samples or standards were loaded into a clear‐bottomed 96‐well plate (SPL, Pocheon‐si, South Korea), and 200 μL of reagent solution was added. The absorbance at 612 nm was measured using a microplate reader (Tecan, Austria). For serum phosphate analysis, 50 µL of 1/20 diluted serum sample, distilled water (blank), or standards were loaded into a clear‐bottomed 96‐well plate, and 100 μL of reagent solution was added. The mixtures were incubated for 30 minutes at RT, and the absorbance at 620 nm was measured using a microplate reader. In addition, the serum levels of osteocalcin (MK127) and CTX‐1 (AC‐06F1) also were analysed in accordance with the manufacturer's protocol.

### 2.12 Statistical analysis

All data are expressed as the mean ± standard deviation (SD). The results are representative examples of at least three independent experiments. Statistical analysis was performed with the unpaired *t* test for two groups and one‐way analysis of variance (ANOVA) for multiple groups; the data showed a normal distribution, and no data points were excluded. *P* values <.05 were considered to indicate statistical significance.

## 3 RESULTS

### 3.1 Identification of PPOAC‐Bz as a candidate disease‐modifying osteoporosis drug effective in the early stages of osteoclastogenesis

**FIGURE 1:**
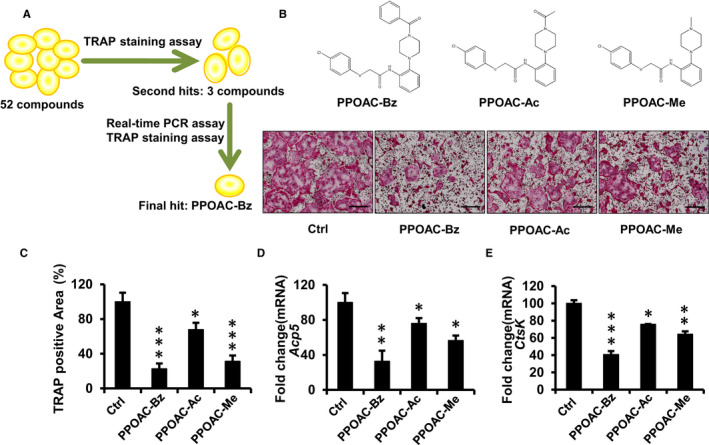
Screening for candidate disease‐modifying osteoporosis drugs (DMOPDs). (A) Schematic diagram of the screening system using TRAP staining and real‐time PCR verification. (B) Structures and TRAP staining of the second hit compounds; scale bar = 200 μm. (C) Counting the spread of the area of osteoclasts after treatment with the second hit compound using ImageJ. (D‐E) Quantification of *Acp5* and *CtsK* mRNA expression in osteoclasts treated with PPOAC‐Bz for 3 d. The fold‐change relative to the vehicle‐treated control is shown. *P < .05, **P < .01 and ***P < .001 vs vehicle‐treated control, (0 μmol/L)

An initial screen was performed among the 52 structurally diverse molecules (Table S2) to select candidate disease‐modifying osteoporosis drugs (Figure 1A). For primary screening, 52 candidate DMOPDs, at a concentration of 10 μmol/L, were screened by performing the TRAP staining assay. After the primary screening, three candidates were chosen as initial hit compounds (Figure 1A,B). To further determine the anti‐osteoclastogenic potential of the three compounds, a TRAP staining assay was performed, and the expression of *Acp5* and *Ctsk* mRNA in osteoclasts was examined after incubation with each of the three compounds (2 μmol/L) for 3 days.

**FIGURE 2:**
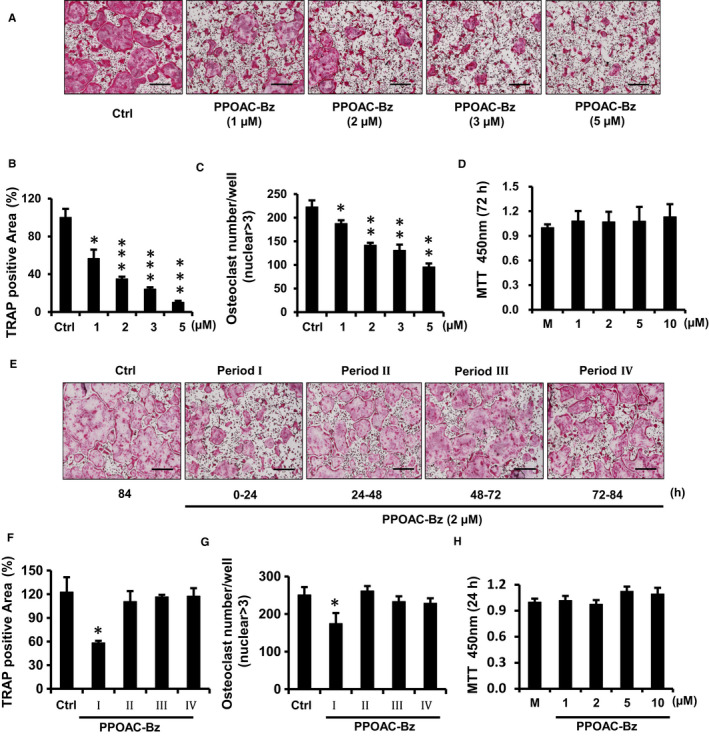
PPOAC‐Bz was identified as a strong inhibitor of osteoclastogenesis that affected the early stages of osteoclast differentiation. (A) The inhibitory effect of PPOAC‐Bz on osteoclast differentiation was observed using a TRAP staining assay. BMMs were treated with various doses of PPOC‐Bz (0, 1, 2, 3, and 5 μmol/L) for 4 d in α‐MEM with M‐CSF and RANKL; subsequently, TRAP staining assays were performed to visualize the formation of mature osteoclasts. The total area (B) and numbers (C) of TRAP+ multinuclear cells were calculated. The MTT assay was performed after 72 h (D). (E) BMMs were divided into five groups (Ctrl, Periods I to IV) and grown in culture medium containing M‐CSF and RANKL for 4 d. The BMMs from the Periods I‐Ⅳ groups were exposed to PPOAC‐Bz for 24 h on different days, respectively. After 4 d, the cells in each group were fixed, and a TRAP staining assay was performed to visualize the osteoclasts. (F‐G) Graphs used for the calculation are shown in Panel (E). The total area (F) and numbers (G) of TRAP‐positive cells were calculated using ImageJ software, and the MTT assay was performed at 24 h (H). *P < .05, **P < .01, and ***P < .001 vs vehicle‐treated Ctrl (0 μmol/L); ‘M’ indicates M‐CSF treatment; scale bar = 200 μm

*N*‐[2‐(4‐benzoyl‐1‐piperazinyl)phenyl]‐2‐(4‐chlorophenoxy)acetamide (PPOAC‐Bz, Figure 1B) was identified as a strong inhibitor of osteoclastogenesis in the TRAP staining assay (Figure 1B,C) and confirmed by real‐time PCR analysis (Figure 1D,E). The area and the numbers of mature osteoclasts were decreased dramatically in the PPOAC‐Bz treatment groups compared with the Ctrl (control) group (Figure 2A‐C), without inducing significant cytotoxicity (Figure 2D), suggesting that PPOAC‐Bz can serve as a candidate inhibitor of osteoclastogenesis. In addition, to confirm whether PPOAC‐Ac and PPOAC‐Me were involved in the late stages of osteoclast differentiation, BMMs were seeded and grown in culture medium containing M‐CSF and RANKL for 48 hours and then exposed to PPOAC‐Ac and/or PPOAC‐Me until the formation of mature osteoclasts was observed in the Ctrl group; subsequently, a TRAP staining assay was performed to visualize the osteoclasts. However, neither of the two compounds showed a significant effect on osteoclast differentiation compared with Ctrl group (Figure S3). To further understand the inhibitory role of PPOAC‐Bz in osteoclastogenesis, 2 µmol/L PPOAC‐Bz was added to the osteoclast induction medium at the indicated time‐points during the process of osteoclast formation. In Figure 2E, the formation of osteoclasts was dramatically suppressed upon treatment with PPOAC‐Bz in the early stages of osteoclast formation (0‐24 hours), without cytotoxicity (Figure 2H); in contrast, no significant difference was observed upon PPOAC‐Bz treatment in the middle stage and late stage (Figure 2E‐G) of formation, suggesting that PPOAC‐Bz inhibited osteoclast formation in the early stages of osteoclastogenesis.

### 3.2 Suppression of RANKL‐induced bone resorption and F‐actin belt formation by PPOAC‐Bz treatment in vitro

**FIGURE 3:**
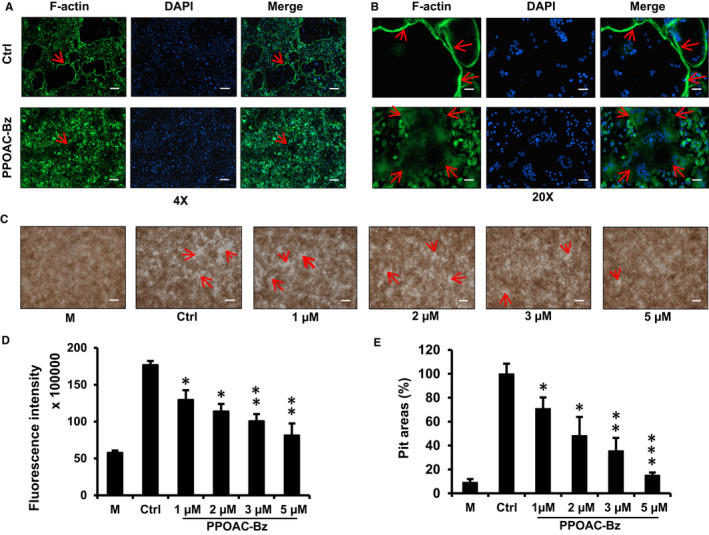
PPOAC‐Bz inhibited the RANKL‐induced formation of F‐actin belts and the activity of bone resorption in vitro. (A) The visualization of the formed and unformed actin belts (4×) during osteoclastogenesis. (B) The visualization of the formed and unformed actin belts (20×) during osteoclastogenesis. (C‐E) BMMs were cultured in a fluorescein amine‐labelled calcium phosphate plate and treated with various doses of PPOAC‐Bz for 6 d. After treatment, the fluorescence intensity in all groups was measured (D). The resorptive pit area was calculated using ImageJ (E), scale bar = 200 μm. *P < .05, **P < .01 and ***P < .001 vs vehicle‐treated Ctrl, 0 μmol/L; ‘M’ indicates M‐CSF treatment; scale bar = 500 μm for 4× images and 100 µm for 20× images

For osteoclast‐mediated bone resorption, the formation of actin belts is regarded as an important visual phenotype of mature osteoclasts. Hence, we performed an immunofluorescence assay to explore the effect of PPOAC‐Bz on the formation of actin belts in RANKL‐induced osteoclastogenesis in vitro. As shown in Figure 3A, the actin rings (arrows) were well formed after supplementation with M‐CSF and RANKL in the ctrl group, whereas the F‐actin belts showed a significant decrease in their sizes and/or formation after treatment with 2 μmol/L PPOAC‐Bz (Figure 3A,B). To explore the in vitro effects of PPOAC‐Bz on osteoclast‐mediated bone resorption, a bone resorption assay kit was used, and the resorptive area was measured using a light microscope (Figure 3C). The results suggested that after M‐CSF and RANKL treatment, the size of the bone resorptive area was markedly enhanced (Figure 3C,E) relative to the negative control, which was only treated with M‐CSF. In addition, the percentage of bone resorption area (arrows) decreased significantly after treatment with 1, 2, 3 and 5 µmol/L PPOAC‐Bz. As shown in Figure 3D, the resorption‐related fluorescence intensity also decreased along with the increase in treatment dose. Together with the effect of PPOAC‐Bz on the formation of mature osteoclasts, these results indicated that the PPOAC‐Bz‐mediated inhibition of bone resorption was attributable to the impairment of mature osteoclast formation and actin belt formation.

### 3.3 PPOAC‐Bz inhibits the expression of osteoclast‐specific markers induced by RANKL

**FIGURE 4:**
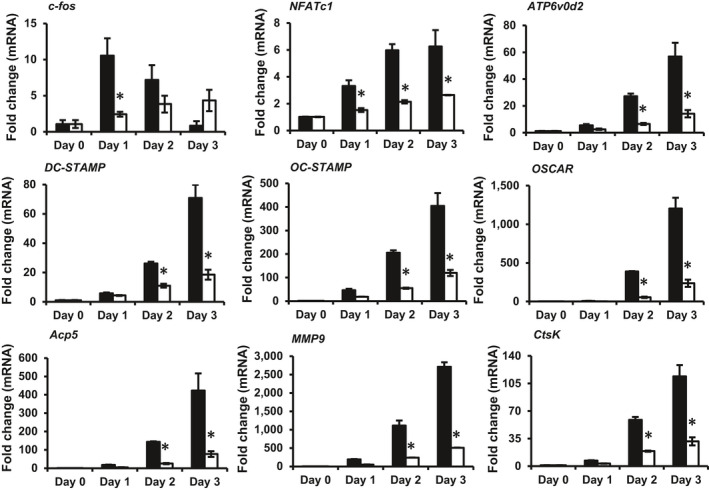
PPOAC‐Bz suppressed the expression of osteoclast‐specific markers induced by RANKL. The relative mRNA expression of the indicated genes was analysed via real‐time PCR after treatment with 2 µmol/L PPOAC‐Bz for 0, 1, 2 or 3 d. Black bars indicate the control, without PPOAC‐Bz treatment, and the white bars indicate the PPOAC‐Bz‐treated groups. The expression of the indicated genes was normalized to the transcript levels of the control on Day 0. Table S1 lists the primers used in this experiment. *P < .05, **P < .01 and ***P < .001 indicate a statistically significant difference between the control and 2 µmol/L PPOAC‐Bz treatment on each day

To explore the role of PPOAC‐Bz in the process of osteoclastogenesis, a real‐time PCR assay was performed. As shown in Figure 4, the mRNA expression of the indicated osteoclast‐specific marker genes increased markedly upon induction via M‐CSF and RANKL treatment, as shown in the control group (black bars). PPOAC‐Bz significantly reduced the RANKL‐mediated transcription of the indicated genes (white bars), which further supported the suppressive effect of PPOAC‐Bz on the formation and function of osteoclasts.

### 3.4 PPOAC‐Bz attenuates the activation of the MAPK and PI3K/Akt signalling pathways in osteoclastogenesis

**FIGURE 5:**
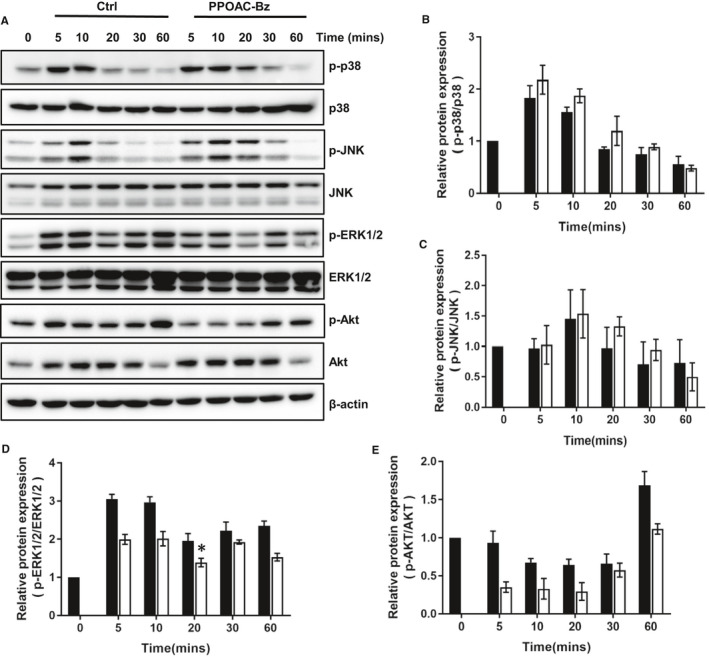
PPOAC‐Bz attenuated osteoclastogenesis by blocking the activation of the MAPK and PI3K/Akt signalling pathways. (A) BMMs were treated with RANKL (100 ng/mL) for the indicated durations of time together with 2 μmol/L PPOAC‐Bz or DMSO, and the levels of phosphorylated p38, JNK, ERK and Akt were analysed using immunoblotting. (B‐E) Densitometry graphs for (A); β‐actin was used as a loading control. *P < .05 vs vehicle‐treated ctrl, 0 μmol/L

The RANKL‐induced MAPK and Akt pathways are necessary for the activation of osteoclasts.^21^ To further examine the mechanisms through which PPOAC‐Bz exerted its suppressive effect on osteoclastogenesis, we examined the influence of PPOAC‐Bz on the MAPK and Akt pathways after co‐incubation with RANKL. The phosphorylation of MAPKs (p‐p38, p‐JNK and p‐ERK1/2), as well as Akt (p‐Akt), as shown in Figure 5A, was determined via Western blotting. Among the MAPKs, the phosphorylation of JNK and p38 did not change significantly; however, compared with the control group, in which phosphorylated ERK1/2 was significantly enhanced upon RANKL stimulation, the phosphorylated form of ERK1/2 was significantly reduced after PPOAC‐Bz treatment. In the case of Akt (Figure 5A), in the control group, p‐AKT was strongly induced when stimulated by RANKL, whereas in the treatment group, Akt phosphorylation was only slightly induced. Collectively, these Western blotting results provided evidence that PPOAC‐Bz could suppress the activation of the MAPK and Akt pathways during RANKL‐induced osteoclastogenesis.

### 3.5 PPOAC‐Bz suppresses osteoclastogenesis by blocking the NFκB and NFATc1 signalling pathways

**FIGURE 6:**
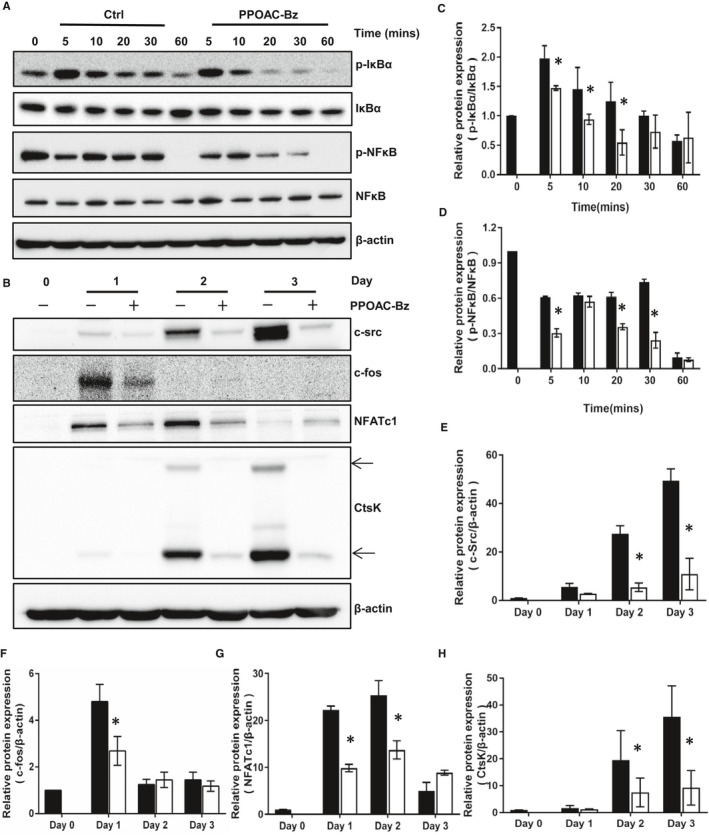
PPOAC‐Bz suppressed the RANKL‐induced activation of the IκBa/p65 (NF‐κB) and NFATc1 signalling pathways. (A) BMMs were treated with RANKL for the indicated durations of time together with 2 μmol/L PPOAC‐Bz or DMSO, and the levels of phosphorylated IκBa and p65 (NF‐κB) were analysed via immunoblotting. (B) BMMs were cultured with 2 μmol/L PPOAC‐Bz for the indicated days in the inductive medium. After the cell lysates were processed, Western blotting was performed with the indicated primary antibodies. (C‐H) The densitometry graphs of (A) and (B); β‐actin was used as a loading control. *P < .05 vs the vehicle‐treated control, 0 μmol/L

In addition to the MAPK and Akt pathways, the RANKL‐induced activation of the NFκB and NFATc1 signalling pathways is an essential step for the differentiation and function of osteoclasts. Mice lacking NFκB, c‐fos and/or NFATc1 can develop osteopetrosis as they are unable to generate mature osteoclasts.^2^, ^14^, ^22^ The activation of NFκB is modulated via four steps: the phosphorylation of IκBα, degradation of IκBα, phosphorylation of NFκB and nuclear translation of the p65 subunit of NFκB.^8^, ^9^, ^23^, ^24^ From our Western blotting analysis, shown in Figure 6A, the phosphorylation of IκBα and NFκB (p65) and the degradation of IκBα were significantly reduced, indicating that the activation of the NFκB signalling pathway was suppressed. Next, we measured the relative expression of c‐fos, NFATc1, the pivotal downstream transcription factors, and CtsK, in the presence and absence of PPOAC‐Bz treatment, as shown in Figure 6B. The protein expression of c‐fos was greatly suppressed on Day 1, and the protein expression of NFATc1 and CtsK was significantly decreased in the PPOAC‐Bz‐treated groups compared with the group not treated with PPOAC‐Bz. These results suggested the suppressive effect of PPOAC‐Bz on the c‐fos/NFATc1 signalling pathway.

### 3.6 Attenuation of OVX‐induced bone loss via PPOAC‐Bz treatment in vivo

**FIGURE 7:**
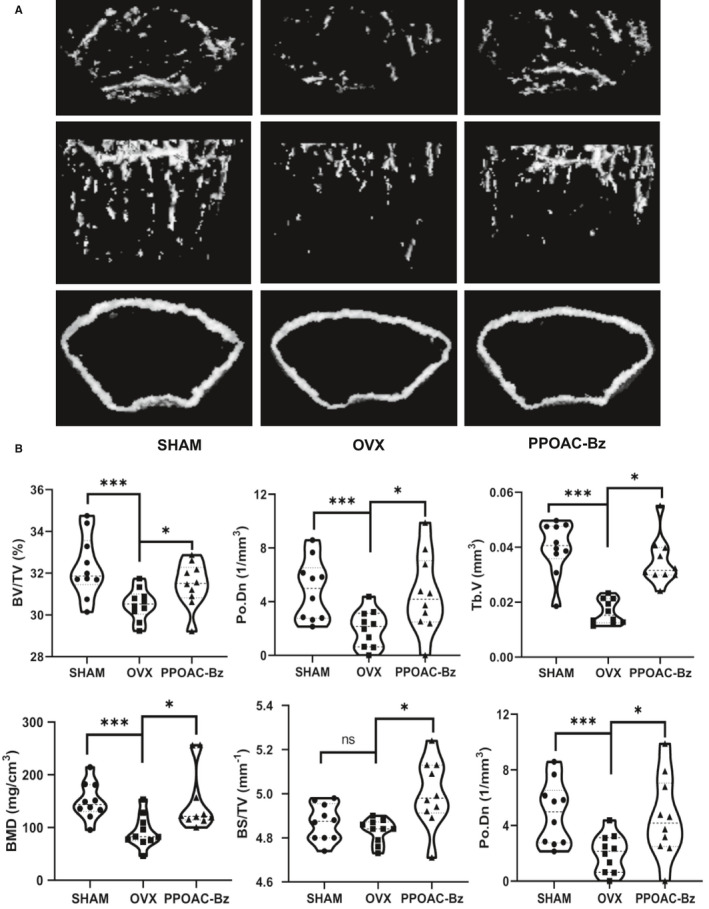
PPOAC‐Bz mitigated the erosion of bone in a mouse model of OVX‐induced bone loss. (A) Representative micro‐CT reconstruction images of mice in the SHAM, OVX and PPOAC‐Bz treatment groups. (B) Micro‐CT analyses of the regions of interest in the femurs. *P < .05, **P < .01 and ***P < .001 vs the control group, OVX

To further investigate the potential preventive effects of PPOAC‐Bz against osteoclast‐related bone‐resorbing diseases in vivo, an OVX mouse model was constructed. After fixing the isolated bones, the whole left femurs and/or tibias of each mouse were scanned using micro‐CT. From the three‐dimensional images of the interested regions of the left femurs and/or tibias, we found that the bone mass in the PPOAC‐Bz‐treated group was not lower than that in the control (OVX group), in the same place, as shown in Figure 7A. The BS/TV, BV/TV, Tb.V, BMD, Po.Dn, and Po.N in the PPOAC‐Bz‐treated groups were higher (Figure 7B) than those in the OVX group. In addition, the femurs were sectioned and subjected to histological analysis using H&E and TRAP staining. The H&E staining results suggested that the remaining trabecular bone was decreased by OVX but was rescued upon PPOAC‐Bz treatment (Figure S1A). The TRAP staining suggested that the amount of TRAP‐positive cells was increased after OVX but decreased after treatment with PPOAC‐Bz (Figure S1B).

To define the biomechanical properties of the bones, the maximum load and slope of the bones were analysed using the three‐point bending test. As shown in Figure S2A,B, in the three‐point bending test, the maximum load was lower in the OVX group than in the sham group; however, the difference was not significant. Interestingly, the PPOAC‐Bz treatment group had a greater maximum load than the OVX group. In addition, there was no significant difference in the slope among the sham, OVX and PPOAC‐Bz treatment groups.

In the serum analysis, serum calcium levels were higher in the OVX and PPOAC‐Bz treatment groups than in the sham group owing to osteoclast‐induced bone resorption (Figure S2C). However, there was no difference between the OVX and PPOAC‐Bz treatment groups in the serum calcium levels (Figure S2C). With regard to the serum phosphorous content, the OVX and PPOAC‐Bz treatment groups showed a higher serum phosphorous content than the sham group. Furthermore, the increased serum phosphorous content was reduced to normal levels upon PPOAC‐Bz treatment (Figure S2D). In addition, the serum analysis of osteocalcin and CTX‐1(Figure S2E,F) showed that OVX significantly increased the serum levels of OCN and CTX‐1; however, PPOAC‐Bz treatment greatly revised the serum CTX‐1, without interference of the OCN level.

## 4 DISCUSSION

The enhanced formation of mature osteoclasts, which accompanies excessive bone resorption, can induce several bone diseases such as rheumatoid arthritis and osteoporosis.^24^, ^25^ Therefore, the prevention or delay of the formation of mature osteoclasts has emerged as one of the main targets for anti‐resorptive drugs.^12^, ^20^, ^24^, ^26^, ^27^ Based on our previous studies^19^, ^21^, ^28^ and the structure of PPOA compounds,^29^ we performed a directed screening of our in‐house compound library, which includes PPOA derivatives, for anti‐osteoclastogenic activity using a TRAP staining assay and a real‐time PCR assay. After screening and further investigations, PPOAC‐Bz was identified as a strong anti‐resorptive agent for the treatment of osteoclast differentiation and exhibited repression of the formation of mature osteoclasts in vitro. Furthermore, PPOAC‐Bz resulted in a noticeable prevention of bone loss in mice with OVX‐induced osteoporosis, as shown by micro‐CT imaging, H&E staining and the bending test of the femur in vivo.

The recruitment of TRAF6, induced by the interaction of RANKL and RANK, can further stimulate MAPK and NF‐κB signalling and/or recruit c‐Src.^20^ Studies have revealed that PI3K/Akt signalling is the downstream pathway of c‐Src recruitment in osteoclast differentiation.^30^ The activation of Akt can further promote the self‐amplification and nuclear translocation of NFATc1 by facilitating the inactivation of GSK3β.^30^ PPOAC‐Bz significantly repressed the activation of Akt, and the expression of c‐Src (Figure 5) was still down‐regulated in the process of osteoclast differentiation, suggesting that PPOAC‐Bz may impact osteoclast formation and bone resorption via repression of the activation of the c‐Src/PI3K/Akt signalling pathway. NF‐κB signalling occurs downstream of TRAF6 during osteoclast formation and has an important role in RANKL‐induced mature osteoclast formation and activation.^23^ The classical NF‐κB signalling pathway suggests that after the activation of the IKK complex, the phosphorylation of IκBα is activated; subsequently, the phosphorylation of IκBα is degraded through an ubiquitin‐proteasome pathway, and NF‐κB transcription occurs.^23^ In the IκB‐independent pathway, IKK can directly phosphorylate NF‐κB, which is able to modulate NF‐κB transcription.^15^ PPOAC‐Bz exhibited an inhibitory effect on the phosphorylation of IκB and the NF‐kB p65 subunit in osteoclastogenesis, indicating that the suppression of osteoclastogenesis by PPOAC‐Bz may be as a result of the inactivation of IκB and NF‐κB. In addition, MAPKs have key roles downstream of TRAF6.^31^ The activation of MAPKs can lead to the translocation of AP‐1, a vital transcription factor for mature osteoclast formation and activation, and subsequently regulate the expression of osteoclast‐related genes such as *CtsK* and *MMP9*, thereby demonstrating a unique role in the process of osteoclastogenesis.^24^, ^25^, ^32^ The MAPK signalling analysis confirmed that the expression of p‐ERK1/2 was dramatically increased in the group with well‐formed mature osteoclasts but was significantly decreased upon incubation with PPOAC‐Bz within 1 hour; in contrast, p‐JNK and p‐p38 were unaffected. It was suggested that RANKL‐mediated MAPKs/activator protein‐1 (AP‐1) and NF‐κB signalling activation occur at a very early stage of osteoclast differentiation.^25^ Our results showed that PPOAC‐Bz could repress mature osteoclast formation only in the early stage, as shown in Figure 2, which was congruent with previous reports.^20^, ^25^ NFATc1, the main transcription factor for osteoclastogenesis,^8^, ^33^, ^34^, ^35^ which was markedly induced in osteoclasts after incubation with RANKL and M‐CSF,^16^, ^25^, ^36^ was markedly down‐regulated upon PPOAC‐Bz treatment, and c‐Src and c‐fos expression was still greatly reduced upon treatment with 2 µmol/L PPOAC‐Bz. Collectively, these data suggested that PPOAC‐Bz mainly suppressed the activation of the NF‐κB and PI3K/Akt signalling pathways and subsequently decreased the formation of mature osteoclasts, which in turn reduced bone resorption activity.

Clinically, denosumab, the first drug targeting osteoclast differentiation, has been approved for the treatment of malignant osteoporosis in both the United States and Europe.^37^ Although it is highly efficacious and there has been a low rate of adverse events in clinical trials, the high cost of the drug has led to further interest in potential alternatives.^37^, ^38^ Besides, other currently available clinical drugs for the treatment of osteoporosis are bisphosphonate and its derivatives; however, the side effects of treatment are jaw and hypocalcaemia osteonecrosis.^17^, ^18^ Thus, novel candidates for the prevention and treatment of osteoporosis are needed. Therefore, we propose PPOAC‐Bz as a potential substitute owing to its effective inhibitory effects on osteoclast differentiation and cost‐effectiveness. Naturally, further exploration and discussion of the novel findings observed in this study are warranted. Bone homeostasis is a complex phenomenon that is related to both osteoclastic bone resorption and osteoblastic bone formation, which are vital events for the treatment of bone diseases.^30^, ^39^, ^40^ However, in the current study, we have focused mainly on an investigation of the inhibitory effects of PPOAC‐Bz on the formation and activation of mature osteoclasts; in further studies, we will evaluate the effect of PPOAC‐Bz on bone formation and the possible mechanism of action.

Collectively, these finding suggest that PPOAC‐Bz attenuated RANKL‐induced osteoclastogenesis by blocking c‐Src expression and NF‐κB and PI3K/Akt signalling. Reductions in the activation of NF‐κB and Akt and the reduction in c‐Src expression mediated the down‐regulation of NFATc1, subsequently leading to a decrease in the expression of osteoclast marker genes. Hence, this study provides proof‐of‐concept that PPOAC‐Bz is a novel inhibitor of resorptive bone loss in mice.

## CONFLICT OF INTEREST

The authors declare no conflict of interest. The funders had no role in the design of the study; in the collection, analyses or interpretation of data; in the writing of the manuscript or in the decision to publish the results.

## AUTHOR CONTRIBUTIONS

**Zhihao Chen:** Formal analysis (lead); investigation (lead); methodology (equal); writing‐original draft (lead); writing‐review & editing (equal). **Eunjin Cho:** Methodology (equal); writing‐review & editing (supporting). **Mina Ding:** Formal analysis (equal). **Jihyoun Seong:** Investigation (supporting); writing‐review & editing (supporting). **Xiangguo Che:** Formal analysis (supporting); methodology (supporting); writing‐review & editing (supporting). **Sunwoo Lee:** Formal analysis (equal); writing‐review & editing (equal). **Byung‐Ju Park:** Conceptualization (equal); project administration (lead); writing‐review & editing (equal). **Je‐Yong Choi:** Writing‐review & editing (supporting). **Tae‐Hoon Lee:** Conceptualization (lead); methodology (equal); project administration (lead); writing‐review & editing (equal).

## Supporting information

Supplementary Material

## Data Availability

The data used to support the findings of this study are available from the corresponding author upon request.
