# Supplementary material for: N‐[2‐(4‐benzoyl‐1‐piperazinyl)phenyl]‐2‐(4‐chlorophenoxy) acetamide is a novel inhibitor of resorptive bone loss in mice
Source: J Cell Mol Med. 2020 Dec 23;25(3):1425–38. doi: 10.1111/jcmm.16228 (PMC7875930; doi:10.1111/jcmm.16228)
Supplement: Supplementary file 1 — Supplementary Material [file JCMM-25-1425-s001.docx]

**Supplementary table 1. Primers used in the study**

| Gene | Primer sequence (5′→3′) | |
| --- | --- | --- |
| *Cathepsin K* | Forward | GGACGCAGCGATGCTAACTAA |
|  | Reverse | CAGAGAGAAGGGAA |
| *Acp5* | Forward | CAGCTGTCCTGGCTCAAAA |
|  | Reverse | ACATAGCCCACACCGTTCTC |
| *DC-STAMP* | Forward | CGCACGATGCTTCATTCTTC |
|  | Reverse | CAGTGCCAGCCGCAATC |
| *GAPDH* | Forward | TGTGTCCGTCGTGGATCTGA |
|  | Reverse | GATGCCTGCTTCACCACCTT |
| *MMP9* | Forward | CTGGACAGCCAGACACTAAAG |
|  | Reverse | CTCGCGGCAAGTCTTCAGAG |
| *NFATc1* | Forward | ACCACCTTTCCGCAACCA |
|  | Reverse | GGTACTGGCTTCTCTTCCGTTTC |
| *ATP6v0d2* | Forward | ACTATGGCCACCCGGGAAT |
|  | Reverse | GGCCCAAGGGAGTCATGTG |
| *c-fos* | Forward | TGGCGGTTTGCACTCTTCA |
|  | Reverse | GGAAGAACTCAGCCAGCTCAA |
| *OC-STAMP* | Forward | TCGGACCCTGGAGGACAA |
|  | Reverse | CCAAACTTGCCAATCTTCCAA |
| *OSCAR* | Forward | GGACCTGTGCTGCCCTAAAG |
|  | Reverse | AGAGAGGACAGGGAGGATCAAGT |

**Supplementary table 2. Compounds used for screening candidate osteoporosis drugs**

| No. | Cat. log | Structure | TRAP+ | No. | Cat. log | Structure | TRAP+ |
| --- | --- | --- | --- | --- | --- | --- | --- |
| 1 | 5117023 |  | 77±7% | 27 | 7919183 |  | 100±20.8% |
| 2 | 5195152 |  | 79±10.2% | 28 | 7937071 |  | 55±13.1% |
| 3 | 5937858 |  | 94±5.4% | 29 | 7959393 |  | 89±9.7% |
| 4 | 6080533 |  | 94±2% | 30 | 7963865 |  | 76±18.8% |
| 5 | 6518758 |  | 110±17.2% | 31 | 7966677 |  | 63±11.2% |
| 6 | 6549908 |  | 91±10.2% | 32 | 7969686 |  | 57±21.8% |
| 7 | 6655846 |  | 76±19.9% | 33 | 7971742 |  | 67±32.9% |
| 8 | 6667142 |  | 107±6.6% | 34 | 7973596 |  | 86±13.6% |
| 9 | 6872009 |  | 74±20.9% | 35 | 7974343 |  | 106±21.7% |
| 10 | 7115624 |  | 53±12.9% | 36 | 7980413 |  | 94±9.8% |
| 11 | 7130083 |  | 79±10.5% | 37 | 7982973 |  | 110±11.8% |
| 12 | 7141820 |  | 71±16.8% | 38 | 7968661 | 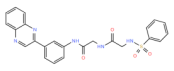 | 32±10.1% |
| 13 | 7241340 |  | 85±29% | 39 | 9070265 |  | 78±32.8% |
| 14 | 7260120 |  | 90±31.2% | 40 | 9074147 |  | 64±5.6% |
| 15 | 7329112 |  | 80±38.7% | 41 | 9138048 |  | 39±16% |
| 16 | 7426869 |  | 96±35.9% | 42 | 9141055 |  | 57±16.6% |
| 17 | 7615424 |  | 68±25% | 43 | 9148746 |  | 59±19% |
| 18 | 7657625 |  | 69±17.3% | 44 | 9148797 |  | 93±28.8% |
| 19 | 7747844 |  | 53±17.1% | 45 | 9150647 |  | 120±0.8% |
| 20 | 7778504 |  | 51±3.5% | 46 | 9158319 |  | 91±30.6% |
| 21 | 7782797 |  | 70±6.5% | 47 | 9296543 |  | 92±14.5% |
| 22 | 7849308 |  | 81±3.8% | 48 | 9309204 |  | 21±19.9% |
| 23 | 7917773 |  | 78±21.7% | 49 | 7933842 | 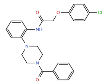 | 22±1.8% |
| 24 | 9272462 | 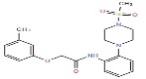 | 75±20.8% | 50 | 9069305 | 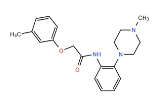 | 100±2.4% |
| 25 | 7932051 | 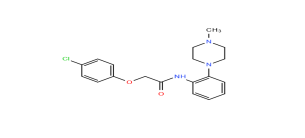 | 32±13.1% | 51 | 9324040 | 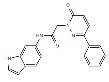 | 100±12.7% |
| 26 | 9021720 | 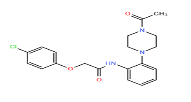 | 28±3.7% | 52 | 9317292 | 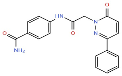 | 97±3.8% |


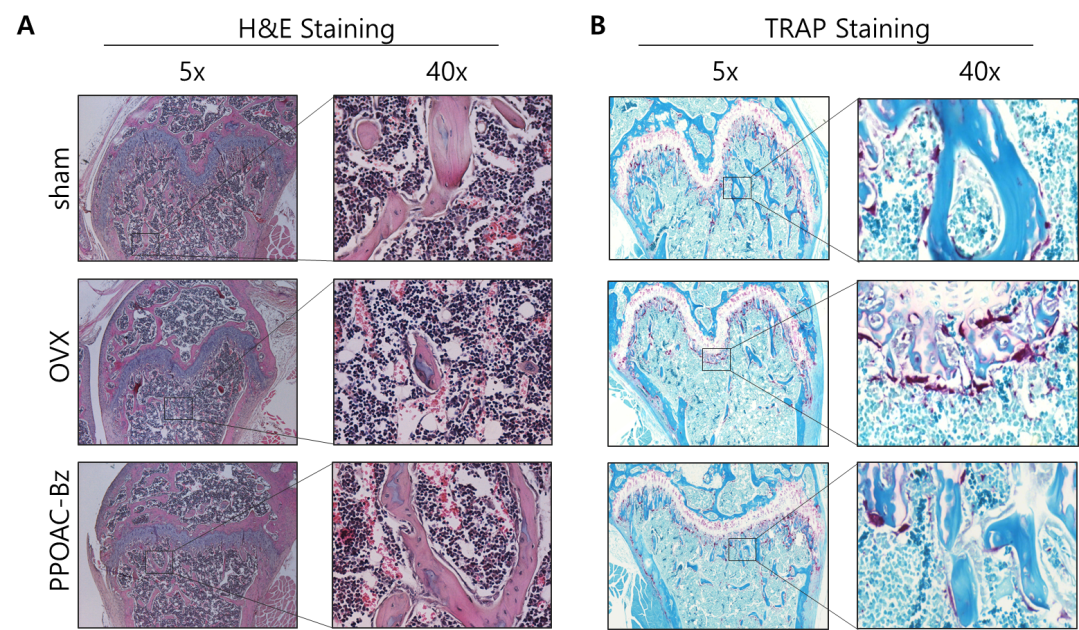


FIGURE S1. Analysis of osteoclast activity *in vivo* in the presence or absence of PPOAC-Bz. Mouse femur sections were fixed, decalcified, and sectioned. (A) H&E staining shows the remaining TB bone in the sham, OVX, and PPOAC-Bz treatment groups. (B) TRAP staining was used to indicate the TRAP-positive cells around the bone (fast green staining was applied to provide a background).


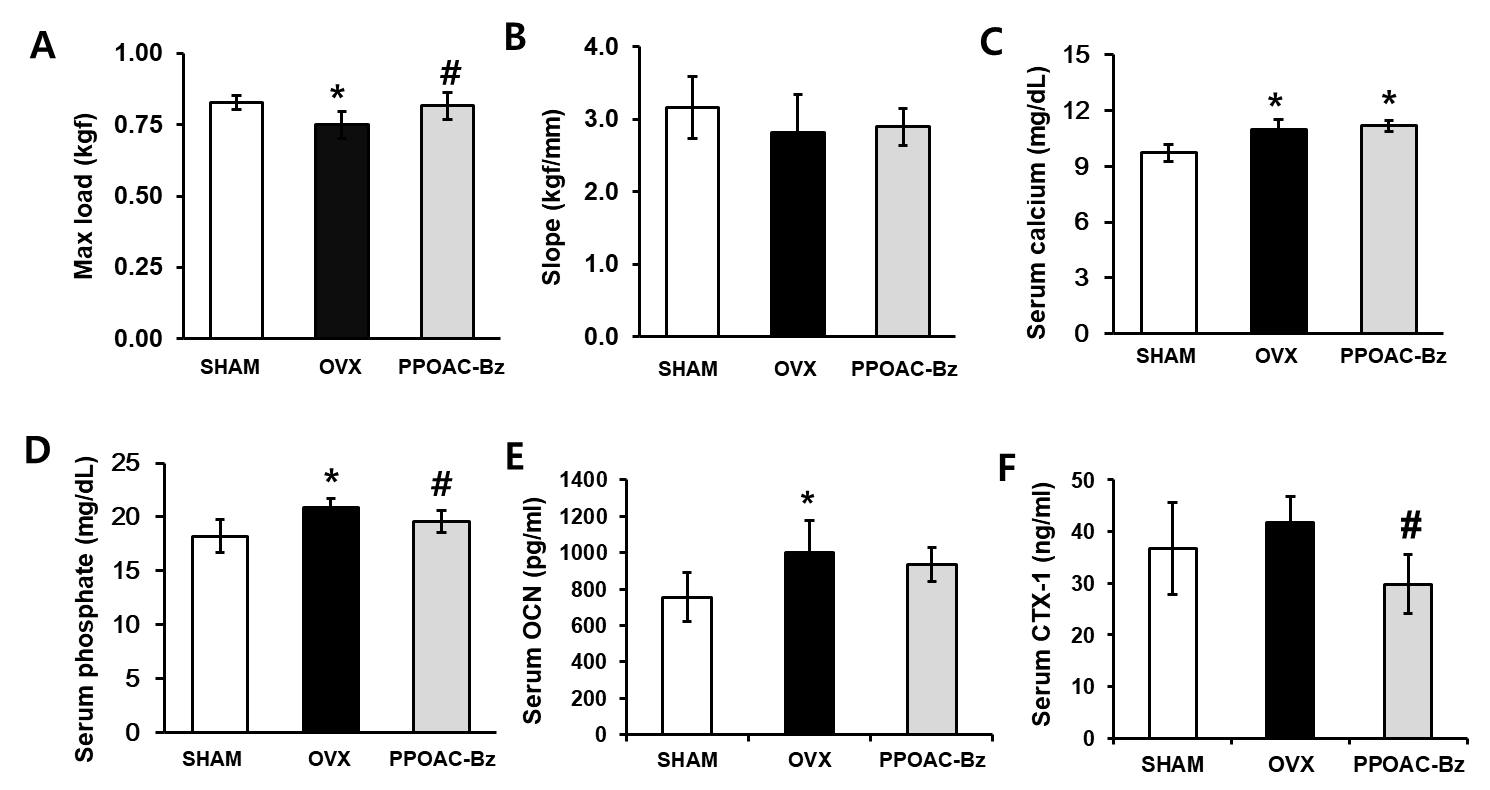


FIGURE S2. The biomechanical properties and serological parameters of femur bones in estrogen deficiency-induced osteoporosis in the presence or absence of PPOAC-Bz. (A) Maximum bending load (kgf), (B) slope (kgf/mm), (C) serum calcium (mg/dL), (D) serum phosphate (mg/dL), (E) serum OCN (pg/mL) and (F) serum CTX-1 (ng/mL) were evaluated for each group. *P<0.05 compared with the sham group; #P<0.05 compared with the control group, OVX.


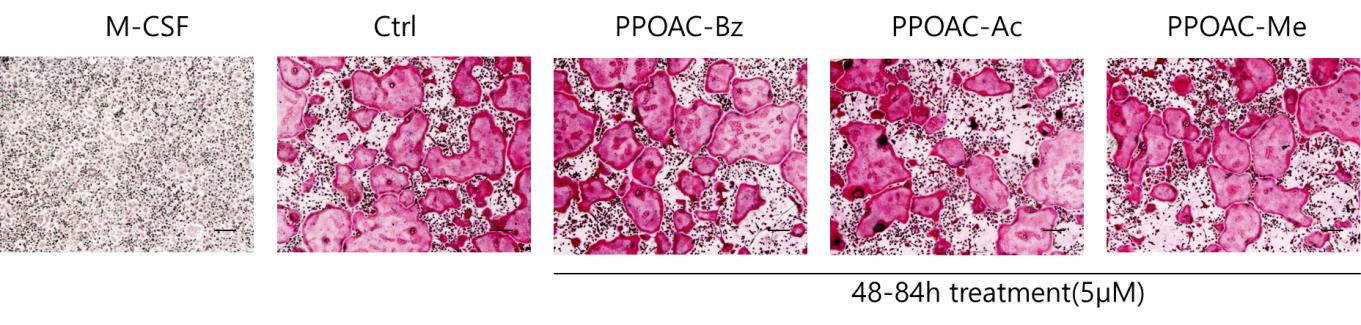


FIGURE S3. Neither PPOAC-Ac nor PPOAC-Me showed a significant effect on the late states of osteoclast differentiation. BMMs were seeded and grown in culture medium containing M-CSF and RANKL for 48 h and then exposed to PPOAC-Bz, PPOAC-Ac, and/or PPOAC-Me until the formation of mature osteoclasts was observed in the Ctrl group. A TRAP staining assay was performed to visualize the osteoclasts. “Ctrl” indicates M-CSF and RANKL treatment; scale bar = 200 μm.
